# Exploring the fitness benefits of genome reduction in *Escherichia coli* by a selection-driven approach

**DOI:** 10.1038/s41598-020-64074-5

**Published:** 2020-04-30

**Authors:** Viktor Vernyik, Ildikó Karcagi, Edit Tímár, István Nagy, Ádám Györkei, Balázs Papp, Zsuzsanna Györfy, György Pósfai

**Affiliations:** 1 Institute of Biochemistry, Biological Research Centre, Szeged, 6726 Hungary; 2 Seqomics Biotechnology Ltd., Mórahalom, 6782 Hungary; 3 HCEMM-BRC Metabolic Systems Biology Lab, Szeged, 6726 Hungary

**Keywords:** Bacterial genetics, Experimental evolution, Genetic engineering, Genomic engineering

## Abstract

Artificial simplification of bacterial genomes is thought to have the potential to yield cells with reduced complexity, enhanced genetic stability, and improved cellular economy. Of these goals, economical gains, supposedly due to the elimination of superfluous genetic material, and manifested in elevated growth parameters in selected niches, have not yet been convincingly achieved. This failure might stem from limitations of the targeted genome reduction approach that assumes full knowledge of gene functions and interactions, and allows only a limited number of reduction trajectories to interrogate. To explore the potential fitness benefits of genome reduction, we generated successive random deletions in *E. coli* by a novel, selection-driven, iterative streamlining process. The approach allows the exploration of multiple streamlining trajectories, and growth periods inherent in the procedure ensure selection of the fittest variants of the population. By generating single- and multiple-deletion strains and reconstructing the deletions in the parental genetic background, we showed that favourable deletions can be obtained and accumulated by the procedure. The most reduced multiple-deletion strain, obtained in five deletion cycles (2.5% genome reduction), outcompeted the wild-type, and showed elevated biomass yield. The spectrum of advantageous deletions, however, affecting only a few genomic regions, appears to be limited.

## Introduction

Genome reduction, contraction of the ancestral gene repertoire, is a natural evolutionary process seen in many bacterial lineages^1–5^. Events leading to genome shrinking are thought to include both neutral and adaptive processes^6–9^.

Artificial genome reduction in laboratory settings, motivated by the search for the minimal life and the corresponding genome, as well as by biotechnological interest, are inspired by the natural evolutionary processes. From the biotechnological aspect, the basic assumption is that bacteria commonly used in the laboratory or industry (e.g., *E. coli*, *B. subtilis*, *C. glutamicum*) carry a wide repertoire of genes, enabling them to survive in their diverse, natural niches. Many of these genes are, however, unnecessary in a narrower regime of conditions in the test tube or fermentor, and the superfluous genes might confer a fitness cost on the host. Elimination of this load would result in cells with improved economy, employing better utilization of the resources. Anticipated benefits include higher growth rate, higher biomass yield, improved genomic stability, and safer handling.

Most of the genome reduction studies targeted *E. coli*^10–15^, but a number of other species have also been subjected to genome streamlining^16–19^. As no direct selection scheme has been put forward for smaller genomes *per se*, the projects were typically based on constructing successive, rationally designed, specific deletions. Targets for elimination were commonly identified by comparative genomics, and mostly included relatively recently acquired, horizontally transferred genomic segments. However, incomplete knowledge of functions and epistatic interactions, as well as possible structural constrains can hamper the effectiveness of design, and laborious construction of individual deletions seriously limit the experimental space of the reduction process. These limitations might be responsible for the mixed results of streamlining. Genetic stabilization of an organism by removal of the mobile genetic elements and error-prone DNA polymerases has been successfully accomplished^20^, and its significance have been demonstrated in *E. coli*^21^. Moreover, occasionally, higher productivity in protein expression^19,22–24^ or small molecule production^14,25^ have been achieved. On the other hand, no real evidence for increased cellular economy has been presented yet. In fact, a gradual decline in growth rate seemed to accompany the progress of genome reduction^26^.

Rather than the maximization of reduction, the aim of the present study was the exploration of the potential benefits of genomic deletions on cellular economy, an analysis made possible by a novel streamlining approach. Instead of rational design, we present here a random, selection-driven procedure, allowing the exploration of a wide range of deletion target choices and alternative reduction courses that give rise to favourable deletion ensembles. Inspiration came from a study^27^ showing that selection can drive gene loss in bacteria.

In the random, iterative deletion scheme (RANDEL), proposed here, any genomic segment can suffer a stimulated deletion in a large population of bacteria, and growth periods inherent in the procedure ensure that cells with the highest fitness can prevail. RANDEL is based on random transposon insertion, followed by infliction of a double-stranded break (DSB) on the transposon, and deletional repair by intrinsic repair mechanisms. The process is aided by antibiotic selection and subsequent counterselection by the dP-hsvTK system. We used a reportedly escapee-free version of the counterselection system, where the herpes simplex virus thymidine kinase (HsvTK), expressed from a duplicated gene, phosphorylates the externally added non-natural nucleoside dP, causing lethal mutagenesis^28^. Applying RANDEL to *E. coli* K-12 MG1655, we show that substantial genome reduction can be achieved without compromising, sometimes even improving the growth properties. However, the choice of advantageous deletions, yielding better economy, appears to be limited to a few genomic regions.

## Results

### Design of the random deletion method (RANDEL)

**Figure 1:**
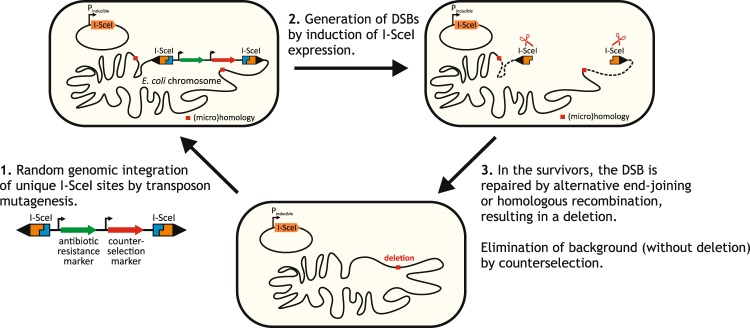
General scheme of the random deletion process. See Fig. 2 for more details.

Our iterative, random deletion method follows the general scheme depicted in Fig. 1. A selectable marker is inserted randomly into the chromosome, followed by the stimulated and selectable eviction of the insertion. Loss of the insertion involves the action of repair mechanisms that remove, in most cases, genomic regions flanking the insertion as well. This in and out process, where both steps can be selected for, ensures that practically all cells will carry a scarless deletion at the end of the cycle.

To efficiently generate an *E. coli* strain library carrying multiple combinations of random deletions, a process fulfilling the following criteria was required: randomness in terms of target location and deletion size, effectiveness approaching one deletion per cycle per cell, scalability to a relatively large population of cells, and robustness against mutational deterioration.

**Figure 2:**
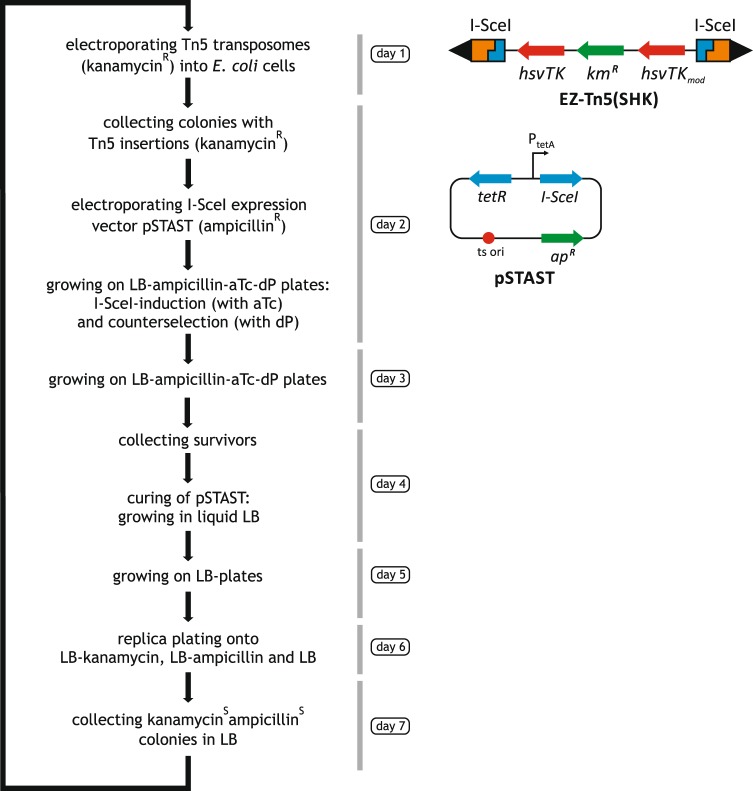
Steps of a random deletion cycle with schematic view of the helper constructs. EZ-Tn5 (SHK) transposon carries a marker for selection (*km*^*R*^) of integrants, *hsvTK* and its modified copy for counterselection, and a pair of I-SceI cleavage sites in opposite direction. pSTAST carries a temperature-sensitive replicon, and expresses I-SceI in an inducible manner. Each cycle incorporates phases of growth (collectively allowing ~90 generations/cycle) during which genotypes with high fitness are selected for.

Steps of the cyclic process are depicted in Fig. 2. Randomness of target location is ensured by employing transposon insertion with nearly no site preference^29^. A modified Tn5 transposon, carrying markers for selection/counterselection, as well as cleavage sites for the meganuclesase I-SceI is inserted in the chromosome. Transposon-inserted cells are selected by their antibiotic resistance. I-SceI, expressed from a plasmid, is then employed to inflict DSBs near the transposon ends, followed by repair of the chromosome by intrinsic mechanisms. Loss of the transposon will be the result of recombination between homologous sequences upstream and downstream of the DSBs mediated either by RecA or the alternative end joining (AEJ) process^30^, using larger homologous stretches (>50 bps)^31^ or microhomologies, respectively. As homologous sequences, especially microhomologies occur throughout the genome, the eventual expanse of the deletion can be regarded as the result of a chance event. Importantly, as AEJ operates rather ineffectively (about 1 in 100 000 DSB-carrying cells are repaired^30^), counterselection must be very potent to eliminate all cells that retained the transposon. Therefore, double counterselection by I-SceI cleavage and the dP-hsvTK system is employed to select survivors that have lost the transposon. Subsequently, the I-SceI-expressing plasmid is cured by a temperature shift, and finally, a replica plating step identifies the cells that are free of the transposon and the helper plasmid.

Scalability is limited only by practical considerations, especially concerning the manual replica plating step. As the ratio of output (cells carrying deletion) and input (transposon-inserted) cells is very low (on par with the spontaneous mutation rate, as indicated in pilot experiments), mutational deterioration of the components of the system (selection/counterselection genes, I-SceI gene, I-SceI target site) is of concern. To prevent this, all components are freshly introduced in the cell in each cycle and, at the end of the cycle, replica plating confirms clearing of the helper components. Importantly, selection of the fittest cells is promoted by the growth periods incorporated in the process (5×/cycle in liquid medium, 4×/cycle on solid medium; collectively allowing ~90 generations of growth per cycle).

### General performance of RANDEL

**Figure 3:**
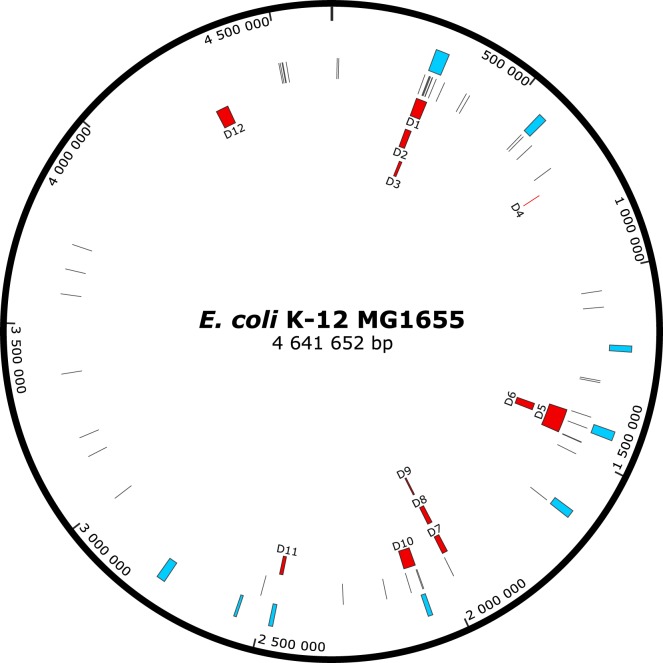
Location of random deletions obtained in the study on the circular genome map. Blue boxes show the position of cryptic prophages, thin lines represent insertion sequences, and red boxes indicate random deletions (D1 to D12).

Effectiveness of the steps of the deletion method was tested in a five-cycle run (Fig. 3). Typically, for each cycle, transposon-inserted cells on the order of 10 000 are obtained in the initial step. Following the counterselection and plasmid elimination, the average ratio of transposon and plasmid loss is around 75%, as revealed by replica plating. Eventually, at the end of each cycle about 500–1000 individual, transposon and helper plasmid free cells are achieved (numbers refer to a protocol easily handled by one person).

The nucleotide analogue dP, used for counterselection, is known to be mutagenic. In our initial protocol, I-SceI cleavage, DSB repair and dP-mediated counterselection progressed simultaneously, and resulted in a high number (1–912/cycle) of point mutations in the surviving genomes (Table 1a). In order to lower the mutational load, the protocol was modified: instead of applying simultaneously, I-SceI cleavage/repair and dP-mediated counterselection were performed sequentially. By giving a 5-h time window to I-SceI cleavage/repair and clearing of HsvTK (the enzyme contributing to dP mutagenesis) from the cell preceding the application of dP, the point mutation rate dropped to background level (0–2 mutations/cycle) (Table 1b). Analysis of the genomes and phenotypic effects below include results obtained using either the highly or weakly mutagenic protocols.

**Table 1:**
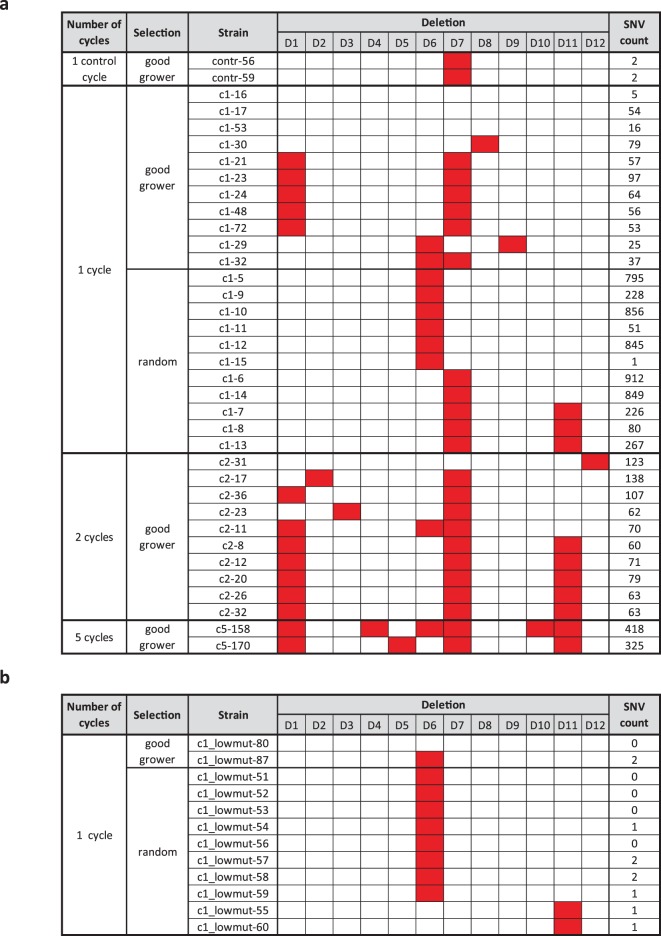
Deletions and background SNV numbers in the strains obtained by the highly mutagenic (a) and weakly mutagenic (b) protocols.

The strains analysed were either pre-selected as good growers (growth parameters not worse than that of the wild-type) or chosen randomly. Red boxes indicate the presence of a particular deletion.

### Deletions and other modifications obtained in the random process

To identify the genetic modifications obtained in the cycles, a total of 60 full genomes were sequenced. These include 2, 11, 10, and 2 cell lines from control (cc), first (c1), second (c2), and fifth (c5) cycles, respectively, all selected as showing good (not worse than that of the wild-type) growth parameters in initial measurements. The genomes of 11 randomly chosen colonies from c1 were also sequenced (Table 1a). In addition, whole genome sequences were obtained from 12 colonies of a single cycle using MDS42 (a strain representing an advanced state of genome reduction) (Table S1, Table S2), and 12 colonies of a single cycle using variants of the protocol with low point mutation load (Table 1b). Growth parameters were re-measured and confirmed individually for all strains selected for sequencing (Table S3).

The number of deletions does not always match the number of cycles (one deletion/cycle). First, in 13% (6/48) of the MG1655-derived strain types, the number of deletions is one short of the expected number. This can be explained by the traceless insertion/excision of the transposon: AEJ repair might proceed via the short homologies at the ends of the transposon insertion. This notion is further supported by the fact that the ratio of the supposed traceless deletions is much higher (75%; 9/12) in MDS42-derived cells, where the purified genome lacks most of the longer repetitive sequences promoting repair. Second, analysis of the first cycle shows a high proportion (29%; 10/34) of cell types possessing one surplus deletion, which is then carried over to the following cycles. This is due to two variants of a deletion (D7 and D8), affecting the flagellar gene cluster that has a high propensity for loss, even without the assistance of DSB/repair, as analysed below.

All together, in single and multiple cycles, 12 different deletions were obtained, ranging in size from 1871 to 70678 bps (Table 2, Fig. 3). Structural requirements for DSB repair seem to be broad: deletion boundaries revealed insertion sequences (IS), phage attachment sites, REP elements, gene-pseudogene pairs, and homologous parts of different genes. The size of these homologies ranged from 11-bp microhomologies to over 1-kbp IS regions (Table 2).

**Table 2:** Features of the deletions obtained in the random deletion cycles. Genomic coordinates refer to the MG1655 genome.

Deletion	Left border	Right border	Size (bp)	Description	Supposed mechanism
D1	258 167	290 892	32 726	genomic segment between two IS1s (incl. the deletion of proline biosynthesis genes and the partial deletion of CP4-6 prophage)	recombination between homologous regions of IS1I and IS1C
D2	258 607	279 861	21 255		recombination between homologous regions of IS1I and IS1B
D3	279 876	291 346	11 471	genomic segment between two IS1s (incl. the partial deletion of CP4-6 prophage)	recombination between homologous regions of IS1B and IS1C
D4	732 605	734 475	1 871	genomic segment between *rhsC* and *rhsO* genes	recombination between homologous regions of *rhsC* and *rhsO* genes
D5	1 397 236	1 467 913	70 678	genomic segment between two IS2 (incl. the deletion of Rac prophage)	recombination between homologous regions of IS2E (its presence at this locus is the result of a duplication) and IS2D
D6	1 411 899	1 434 958	23 060	Rac prophage	excision at the attachment site of Rac
D7	1 962 083	1 978 502	16 420	flagellar and chemotaxis genes flanking IS1	recombination following the replicative transposition of IS1H
D8	1 962 204	1 978 502	16 299		
D9	1 972 841	1 978 502	5 662		
D10	2 066 704	2 102 294	35 591	genomic segment between two IS5 (incl. the deletion of histidine biosynthesis genes and CP4-44 prophage)	recombination between homologous regions of IS5H and IS5I
D11	2 466 369	2 476 583	10 215	CPS-53 prophage	recombination between homologous regions of *argW-pawZ* gene-pseudogene pair at CPS-53 attachment site
D12	4 285 317	4 323 260	37 944	genomic segment between REP elements	recombination between homologous regions of REP320 and REP324

In the sequenced 34 single-cycle genomes, only 6 different ones were observed. Two of these affected cryptic prophages (Rac, CPS-53), and both of them occurred more than once among the sequenced genomes. Interestingly, deletion of the flagellar genes appeared multiple times in the first cycle, and occurred even in the control cycle (without I-SceI cleavage). We tested our initial stock of MG1655, used for the five-cycle run, and found that 25% of the cells had already lost this genomic region. Further tests showed that these cells, having a competitive advantage, are enriched in this population from 25% to 75% in a single control cycle.

Use of the initial, highly mutagenic protocol resulted in tens of single nucleotide variants (SNVs) per cycle per genome on average, and the two sequenced genomes emerging from the fifth cycle carried hundreds (418 and 325) of SNVs. In addition to SNVs, a large duplication was also observed in one of these genomes (Fig. S1).

Distribution of the SNVs (Fig. S2) provides information on the independent or common origin of the cell lines. Similar SNV pattern in 4 first-cycle, and 5 second-cycle genomes among the “good growers” suggest selection of these advantageous deletional variants. Differing SNV patterns reveal that the multiply occurring deletions, prophages Rac (D6), CPS-53 (D11) and, prominently, the flagellar region (D7, D8, D9), emerged independently as well. Taken together, evidence suggests that some genomic regions have a high propensity for loss driven by selection, and beneficial deletion-SNV combinations are quickly fixed in the populations.

### Delimiting the phenotypic effects of deletions and point mutations

**Figure 4:**
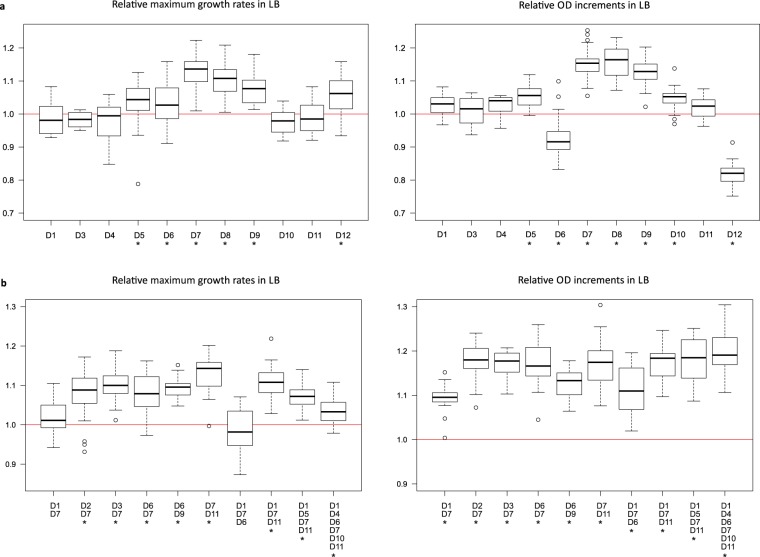
Growth parameters of single- (**a**) or multiple-deletion (**b**) strains with reconstructed deletions. Strains are identified by the particular deletions (D1 to D12) they carry. Maximum growth rates and OD increments were compared to those of wild-type MG1655. Values are based on at least 18 replicate measurements in rich medium (LB). Centre lines show the medians, box limits indicate the 25th and 75th percentiles, whiskers extend 1.5 times the interquartile range, and outliers are represented by circles. Asterisks indicate significant difference in comparison to the wild-type (Welch’s t-test, P < 0.05).

To analyse the impact of deletions without the interference of the unwanted mutations, individual deletions and their combinations, obtained in the five-cycle run by the mutagenic procedure, were reconstructed in the clean background of the non-evolved, mutation-free parental host. Growth rate and OD increment (a proxy for biomass yield) data of the strains are depicted in Fig. 4. While some of the deletions did not change the growth parameters, six deletions (50% of all deletions), essentially representing three types (flagellar apparatus, Rac, D12) caused a significant elevation of the growth rate. Particularly strong effect was seen when the deletion disabled the flagellar apparatus. Even when combined with five additional deletions, the multideletional strain showed elevated growth rate and biomass yield, compared to wild-type. All variants of the flagellar deletions resulted in a non-motile phenotype.

The number of SNVs in the sequenced genomes varied wildly (0-912/cycle, and up to 418 after 5 cycles). Comparison of the same deletional variants possessing various number of accompanying SNVs (e.g., zero SNV in reconstructed genomes, and various number of SNVs in independent lineages carrying the same deletion) revealed a tendency: while a low mutation rate allowed the occasional emergence of mutations increasing the fitness, a high mutational load, due to the effects of the overwhelmingly deleterious SNVs, caused a decline in growth parameters (Fig. S3). Moreover, strains emerging from the highly mutagenic protocol showed a rapid decline in viability in another niche, glucose minimal medium (Fig. S4b).

Interestingly, reconstructed “clean” deletion of Rac prophage caused a drop in the yield, but this loss was not observed in the Rac deletion strain isolated by the random deletional cycle, where a single SNV in *yehB* accompanied the deletion. By recloning this point mutation in the “clean” deletion strain, we proved its compensating effect (Fig. S5).

Selected single and multiple deletion strains were tested in competition assays against parental MG1655 (Table 3). Importantly, the reconstructed strain with the highest number of deletions (6 deletions, including the flagellar one), having a genome reduced by 2.5%, outcompeted the wild-type strain in LB medium (Table 3). Biomass yield (dry mass) of this strain showed 6% elevation, compared to MG1655 (Table 4).

**Table 3:** Competitive fitness of deletion strains compared to the wild-type MG1655 ancestor.

Strain	Competitive fitness relative to wt	P value
D6	1.00 (0.008)	0.5031
D7	1.11 (0.030)	0.0243
D1_D4_D6_D7_D10_D11	1.03 (0.006)	0.0028
D1_D4_D6_D7_D10_D11 (c5-158)	0.92 (0.009)	0.0043

Strains are identified by the particular deletions they carry. D6, D7, and D1_D4_D6_D7_D10_D11 are reconstructed deletion strains with clean parental genetic background. Strain D1_D4_D6_D7_D10_D11 (c5-158) was obtained by the mutagenic, random procedure. Mean and standard deviation values (in parentheses) are based on at least 3 independent experiments. Competition assays were performed in rich medium (LB). One-sample t-tests showed that D1_D4_D6_D7_D10_D11 (c5-158) has significant competitive disadvantage, while D7 and D1_D4_D6_D7_D10_D11 have significant competitive advantage over the wild-type (P < 0.05).

**Table 4:** Biomass yield of the reconstructed 6-deletion strain and the wild-type MG1655 ancestor.

Strain	Dry mass (g/l)
MG1655	1.83 (0.034)
D1_D4_D6_D7_D10_D11	1.95 (0.064)

Dry mass of 20-ml overnight shake-flask cultures grown in rich medium (LB) at 37 °C was determined. Mean and standard deviation values (in parentheses) are based on 5 replicate measurements. Welch’s t-test showed significantly higher biomass yield of the reconstructed 6-deletion strain D1_D4_D6_D7_D10_D11 (P = 0.011).

The streamlining process was carried out in rich medium. Favoured deletions, selected in the procedure, might come with a trade-off: cells carrying deletions D1, D2, and D10 are non-viable in minimal medium, due to the loss of genes involved in amino acid synthesis (Table 2).

### Comparison of deletions obtained by targeted and random methods

Our earlier, published genome reduction work was driven by rational design^13,26,32^. Selection of genomic regions targeted for elimination was based on comparative genomics, as well as on available gene essentiality and functionality studies. Interestingly, comparison of the 12 targeted deletions (totalling 423 genes/376 kbps) of the milestone strain MDS12^32^ with the 12 random deletions obtained here (totalling 218 genes/206 kbps) reveals only 7 matching cases, underscoring the differences in the preferences of rational design and selection-driven random streamlining (Fig. S6). The extended deletion set of the advanced milestone strain MDS69 (69 targeted deletions, totalling 965 genes/942 kbps)^26^ largely encompasses all our 12 random deletions (Fig. S6), but there are some differences in the extension of the corresponding eliminated segments. All together, relative to MDS69, deletion of 43 genes are novel in the random set.

**Figure 5:**
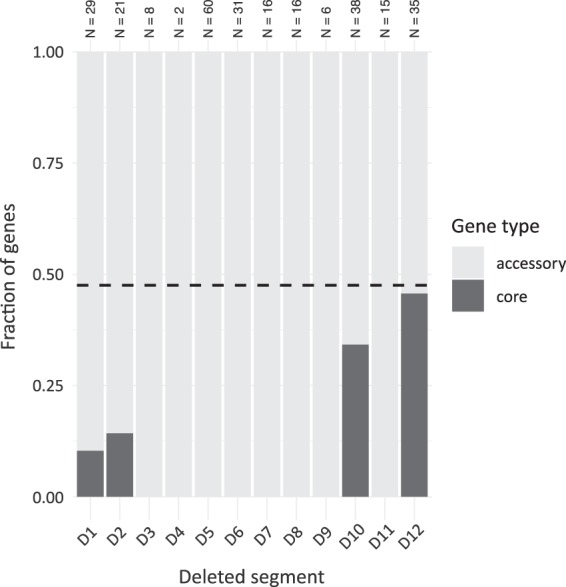
Frequencies of core and accessory genes within the deleted segments. Identities of the particular deletions (D1 to D12) are given in Table 2. Dark bars represent the fraction of core genes compared to the total number of genes residing in the particular deleted region. The total number of genes that can be classified into core and accessory within each deleted region are shown on top of the bars. Dashed line represents the fraction of core genes in the entire genome.

Finally, we asked whether the randomly deleted segments are devoid of genes that are part of the *E. coli* core genome (i.e., genes present in all strains within the *E. coli* clade, see Methods). As expected, most deleted segments contained zero or only a few core genes (Fig. 5), suggesting that our approach tend to target accessory genes. Strikingly, however, we found two regions, D10 and D12, that contain core genes with frequencies almost as high as the genomic average (Fig. 5). This finding suggests that our selection-driven genome reduction approach can also target regions that would not be predicted based on comparative genomic approaches.

## Discussion

RANDEL allows the efficient generation of successive random deletions in the *E. coli* genome, accompanied by simultaneous selection of the fittest variants in the population. The time-frame, labour intensity, and rate of genome reduction (average deletion size was 24 kbp) are comparable to those of the targeted methods^33^. We did not invest in optimization of the steps, but anticipate that the time-frame of the cycles (~one deletion/week) can be substantially shortened. Besides the deletions, the initial protocol generated SNVs as well, due to the application of the mutagenic counterselection agent dP. Modification of the protocol resulted in low, basically normal background mutation rate. Replacing the hsvTK-dP system by other, highly effective non-mutagenic counterselection method^34,35^ might also provide alternatives.

In the sequenced 60 genomes, only 12 different deletions (some of them occurring multiple times) were observed. This limited variability of deletions in the initial cycles can be explained by three factors: (i) specific mechanisms promoting deletion of the particular segment (e.g., stress-induced excision of the prophage by its own system), (ii) deletion hotspots due to extended homologous sequences, and (iii) quick selection/elimination of a particular deletion due to the competitive advantage/disadvantage it confers. Literature data and our own results provide support for all three mechanisms. Excision of cryptic *E. coli* prophages at low frequencies has been described^36^. Here, stress imposed by dP treatment might stimulate excision. In addition, 200-fold differences in deletion frequencies at different loci have been reported^27^. Spontaneous deletion of the flagellar genes at a high rate by a proposed IS-mediated mechanism, followed by its rapid fixation has also been described^37^.

Selection-driven gene loss has been reported in *E. coli*, albeit deletions affecting only two genomic regions have been shown to provide competitive advantage^27^. Similarly, in LB selection medium, we find fitness-increasing deletions, but in limited numbers, affecting the flagellar cluster, the Rac prophage (with or without an adjoining region), and a 38-kbp genomic segment (D12). The other deletions seem to be largely neutral, riding along the beneficial ones, at least under the tested growth condition. Future works should explore whether further fitness-increasing deletions can be identified by RANDEL when growth periods are carried out in different environmental conditions^9^.

The reason behind the frequent loss of the flagellar apparatus, not needed in the shake flask, is obvious: the estimated cost of running flagella is 4.5% of the cellular energy^27^. Deletion of this energy-consuming structure increases both the growth rate and biomass yield. Competitive advantage of this strain in the shake-flask cultures is significant, even when five additional, mostly neutral deletions are carried in the genome. Advantage of the other fitness-increasing deletions is not obvious. As the missing DNA represents negligible energetic gain^38^, ceasing synthesis of unnecessary proteins might be the clue.

Interestingly, most of the deleted segments appear to be part of the accessory genome of the *E. coli* clade. These genes might play roles under specific conditions only (e.g., flagellar genes) or might have been acquired recently through horizontal transfer (e.g., prophages) and are more amenable for loss under lab conditions. On the other hand, we found some deletions that clearly carve into the core genome. Notably, the dispensability of such regions couldn’t be predicted based on comparative genomic approaches.

Laboratory evolution has been successfully applied to improve the fitness of a reduced-genome *E. coli* strain^39^. However, this post-reduction evolution does not allow the selection-aided probing of multiple, potentially beneficial deletional trajectories. In contrast, RANDEL allows the experimental exploration of a wide space of evolutionary tracks of streamlining, accompanied by the emergence of adaptive and compensating small mutations. Obtaining a multiple-deletion strain (6 deletions, a total of 2.5% genome reduction) displaying increased competitive fitness and elevated biomass yield demonstrates the potential of the approach. On a longer run, even if fitness-increasing deletions seem to be of limited number, significant genome simplification might be achieved by iteratively accumulating largely neutral deletions, meanwhile selection picking the variants with the best fitness. In the process here, we used rich medium to select for the fittest variants, but growth periods under other conditions suited for specific goals can be incorporated to serve as evolutionary filters.

## Conclusions

In contrast to targeted methods, the iterative random deletion procedure presented here enables the exploitation of multiple genome streamlining trajectories and involves the automatic selection of the fittest variants in the population. We showed that elimination of certain genomic segments can improve competitive fitness and biomass yield, but the choice of the truly beneficial deletions are limited to a few genomic regions. Nevertheless, the process allows the accumulation of deletions in favourable combinations, without a priori knowledge of the gene network.

## Methods

### Strains, plasmids and media (Table S4)

*E. coli* K-12 strain MG1655^40^ (NC_000913.3), its multideletional derivative MDS42^13^ (NC_20518.1) and MDS42π (expressing the Pir protein) were used. MDS42π was constructed by carrying over *pir* from DH5α*pir*^41^ to MDS42 by P1 transduction. DNA constructs were created in MDS42, random deletion cycles were performed both in MG1655 and MDS42. MDS42π strain was used for maintenance of plasmids with R6K γ origin of replication (e.g., pSG76-A). pHKH^28^ and pSG76-A^42^ served as PCR templates for transposon construction. pSTAST^32^ plasmid was used for induced expression of I-SceI. pST76-A^42^ based constructs were applied for generation of targeted deletions. In all experiments involving bacterial culture growth, standard LB medium or MS-minimal medium^43^ with 0.2% glucose were used. For recovery after electroporation, SOC medium was applied. MacConkey agar plates were used for differentiating cells in competition experiments.

The inducer anhydrotetracycline (aTc) was used at 100 ng/ml, the nucleoside analogue 6-(β-D-2-deoxyribofuranosyl)-3,4-dihydro-8H-pyrimido-[4,5-c][1,2]oxazin-7-one (dP) was applied at 1 μM final concentration. Antibiotics were used at the following final concentrations: 100 μg/ml ampicillin (ap), 25 μg/ml kanamycin (km).

### Construction of the modified EZ-Tn5 transposon and transposome preparation

pSG76-A was linearized by PCR with primers containing overhangs with hyperactive Tn5 mosaic ends and I-SceI sites. The selection cassette (*hsvTK-km*^*R*^*-hsvTK*_*mod*_) was PCR-amplified from pHKH and ligated with the plasmid backbone including mosaic ends and I-SceI sites resulting in pSG76-A-Tn5-SHK. The transposon EZ-Tn5(SHK) (Fig. S7) was cut from the plasmid with *PvuII*. To generate transposomes^44^, the gel-isolated fragment was incubated with EZ-Tn5 Transposase (Lucigen) according to the manufacturer’s instructions. pSG76-A was chosen as a plasmid backbone for its origin (R6K γ-ori) does not allow replication in target cells.

### Random deletion cycles

Steps of a random deletion cycle are depicted in Fig. 2. Electrocompetent cells were prepared from the parental strain or from cells obtained at the end of the previous cycle. In a typical experiment, 0.5 μl of EZ-Tn5(SHK) transposome was electroporated into 40 µl of electrocompetent cells, spread onto LB-km plates and incubated overnight at 37 °C. The insertion mutant colonies were washed off in LB-km, diluted to OD_550_~1 in 10 ml LB-km, grown for 2–3 hours at 37 °C and used as starter for making electrocompetent cells. ~50 ng of pSTAST was electroporated into 40 µl of electrocompetent cells, and spread onto LB-ap-aTc-dP plates (aTc was applied for I-SceI induction, dP was used for counterselection). Plates were incubated at 30 °C for 35–40 hours. For curing the helper plasmid, colonies were washed off in LB, and diluted in in 10 ml LB to OD_550_~0.1 and grown overnight at 37 °C. Dilutions were spread onto LB plates and after overnight incubation at 37 °C, colonies were streaked onto LB and LB-ap/km to select cells that are sensitive for both km and ap (lost both the transposon and the helper plasmid). The selected colonies were inoculated into 10 ml LB for starter used in the next cycle. For the weakly mutagenic version of the protocol, the following modifications were made: after the pSTAST-electroporation, cells were transferred into 10 ml LB-ap-aTc and shaken for 5 hours at 30 °C to induce I-SceI. Aliquots were spread onto LB-ap-aTc-dP plates and incubated at 30 °C for ~16 hours. The rest of the cycle was the same as described above.

### Strain construction

Targeted, re-constructed deletions were constructed in MDS42 host by a suicide plasmid-based deletion method, as previously described^45^.

## Genome sequencing

Ion Torrent library construction was performed as described previously^46^. The sequencing data was processed using Ion Torrent Suite v5.2 in order to perform signal processing and base calling. Raw reads were imported into CLC Genomics Workbench v11.0 (Qiagen), trimmed and mapped to the *E. coli* K12 substr. MG1655 (NC_000913.3) or MDS42 genome sequence (NC_020518.1). Variant regions were manually inspected in all strains. Large genomic rearrangements (typically deletions with length above 1 kb) were manually identified using CLC Genomics Workbench Tool.

### Measurement and calculation of growth parameters

To measure growth parameters, cells were grown in Synergy 2 automated microplate reader machine (BioTek). 1 μl from overnight starter cultures were transferred into 99 μl fresh LB medium or MS-minimal medium with 0.2% glucose in 96-well plates. For each strain, OD_600_ was measured every 5 minutes for 24 hours at 37 °C with continuous shaking. Growth parameters (growth rate, OD increment) were calculated by using previously described methods^47^. 4 to 18 replicates were used for each strain.

### Competition of strains

Overnight starter cultures of the wild-type MG1655 and the competing strain were mixed at a 1:1 volumetric ratio, then 40 μl of the starter mix was inoculated into 10 ml LB medium and grown in a 100-ml Erlenmeyer flask with shaking at 225 rpm at 37 °C for 24 hours. 40 μl of the culture was then transferred daily (up to 3 days) into fresh medium. For each strain, experiments were replicated 3–4 times. To track the progress of competitions, samples were taken immediately after inoculation and at every re-inoculation of cultures. A LacZ^-^ variant of MG1655, where the LacZ activity was inactivated by incorporation of a nonsense mutation^48^ was used for differentiating the competing strains. Cell counts of each competitor were determined by spreading appropriate dilutions on MacConkey agar plates. Relative fitness values were estimated as described^49^. The natural logarithm of the ratio of the competing and the parental strain was plotted against the number of generations, where the slope of the linear regression line is the selection coefficient (s) of a given strain. Competitive fitness (w) values relative to the parental strain were calculated as 1 + s.

### Motility assay

To test bacterial motility, cells were stabbed into 200 μl soft agar (LB medium containing 0.3% agar) dispersed into wells of 96-well plates. Following overnight incubation at 37 °C, motility was observed visually as diffuse growth from the site of inoculation.

### Measurement of biomass yield

Biomass yield was measured in LB, the medium used throughout the streamlining process. Exponential-phase starter culture was inoculated into 20 ml LB medium with initial OD_550_ = 0.01 and grown in a 100-ml Erlenmeyer flask with shaking at 160 rpm at 37 °C overnight. 8 ml of culture was pelleted in microcentrifuge tubes and the pellet was dried in a ScanSpeed 40 vacuum centrifuge (1000 rpm, <0.3 mbar) for 20 hours. The mass of tubes was measured on an analytical scale. To obtain the dry biomass, the mass of empty tubes was subtracted from the mass of each tube containing dried pellet. The measurements were performed in 5 biological replicates per strain with 2 technical replicates per culture.

### Core genome analysis of the deleted segments

Genes residing in the D1..12 segments were classified either as core or accessory based on a previous study, which identified ~2000 core genes that are shared between 60 fully sequenced *E. coli* genomes^50^. Some genes were unclassified and these were manually inspected using functional information from the UniProt database. Insertion elements, prophages and genes with unknown functions were classified as accessory, while the rest were excluded from the analysis.

## Supplementary information

Supplementary Information.
