# Supplementary material for: Exploring the fitness benefits of genome reduction in Escherichia coli by a selection-driven approach
Source: Sci Rep. 2020 Apr 30;10:7345. doi: 10.1038/s41598-020-64074-5 (PMC7193553; doi:10.1038/s41598-020-64074-5)
Supplement: Supplementary file 1 — Supplementary Information. [file 41598_2020_64074_MOESM1_ESM.pdf]

Exploring the fitness benefits of genome reduction in *Escherichia coli* by a selection-driven approach

Viktor Vernyik, Ildikó Karcagi, Edit Tímár, István Nagy, Ádám Györkei, Balázs Papp, Zsuzsanna Györfy, György Pósfai

Supplementary information

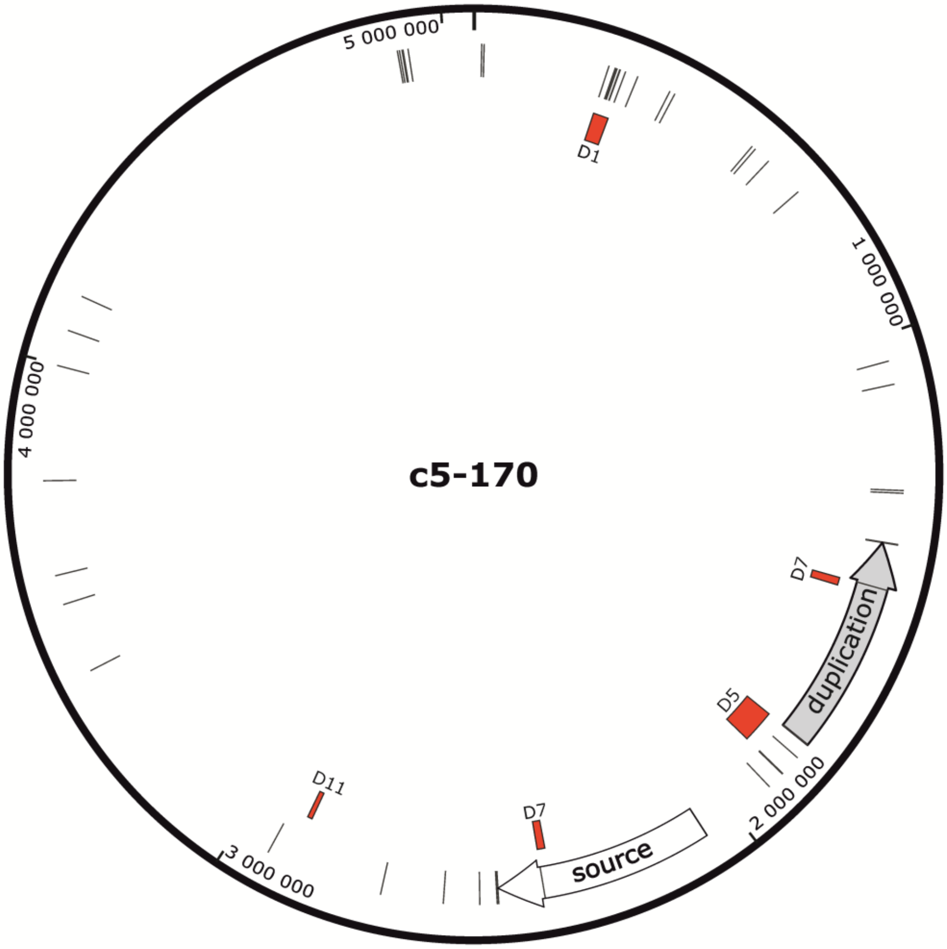

Figure S1. Location of the duplication in the random deletion strain from the fifth cycle (in strain c5-170). The genome map of wild-type MG1655 is extended with the 398-kb long duplication, indicated with grey arrow. The source of the duplicated region is shown with white arrow. Thin lines represent insertion sequences, and red boxes show random deletions (D1, D5, D7, D11).

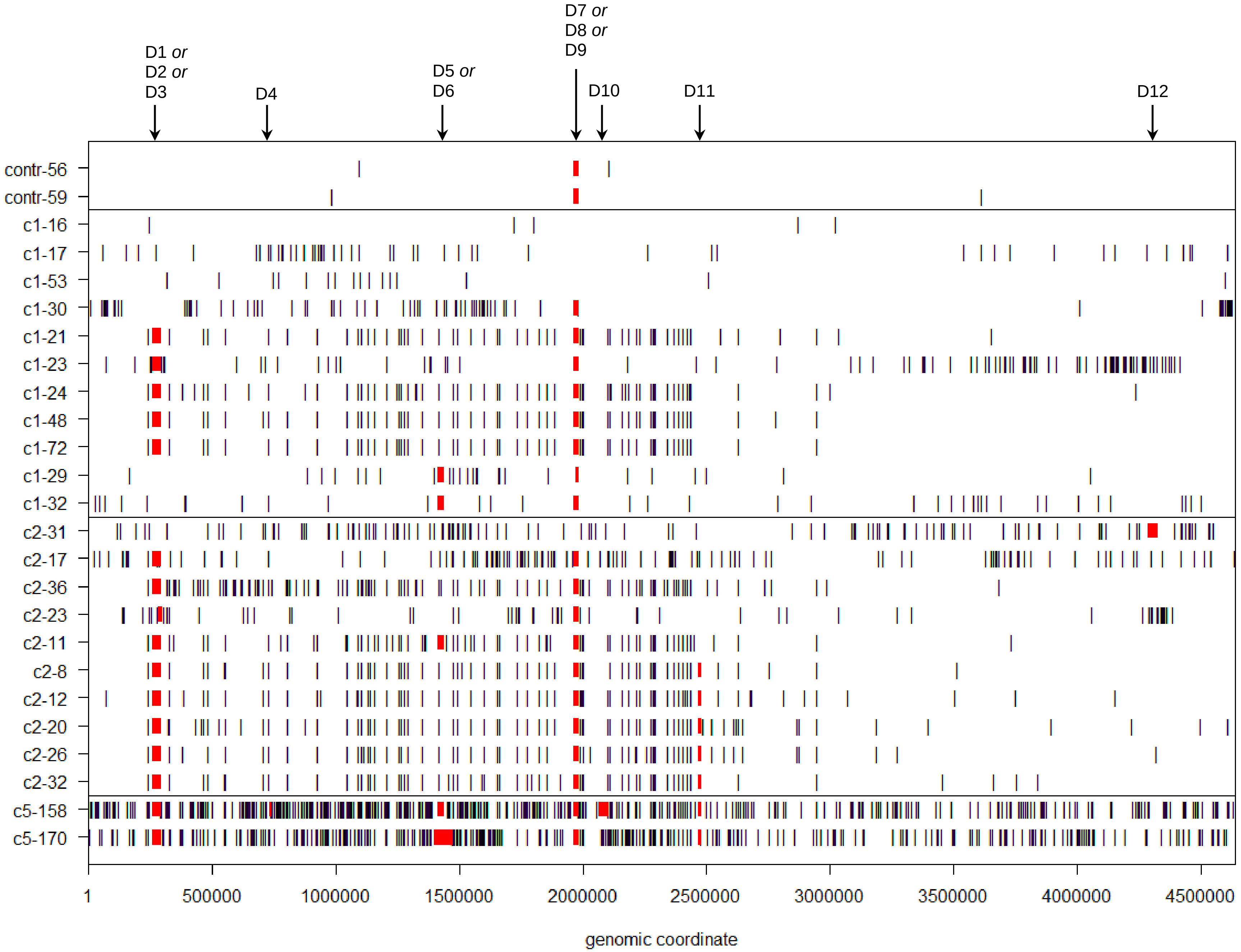
D1 *or*
D2 *or*
D3
D4
D5 *or*
D6
D7 *or*
D8 *or*
D9
D10
D11
D12
contr-56
contr-59
c1-16
c1-17
c1-53
c1-30
c1-21
c1-23
c1-24
c1-48
c1-72
c1-29
c1-32
c2-31
c2-17
c2-36
c2-23
c2-11
c2-8
c2-12
c2-20
c2-26
c2-32
c5-158
c5-170
1
500000
1000000
1500000
2000000
2500000
3000000
3500000
4000000
4500000
genomic coordinate

Figure S2. Distribution of SNVs in the genomes of the control strains (carried through a single cycle without transposon insertion), and deletion strains (obtained by the mutagenic procedure). Red boxes represent deletions D1 to D12, vertical lines indicate SNVs.

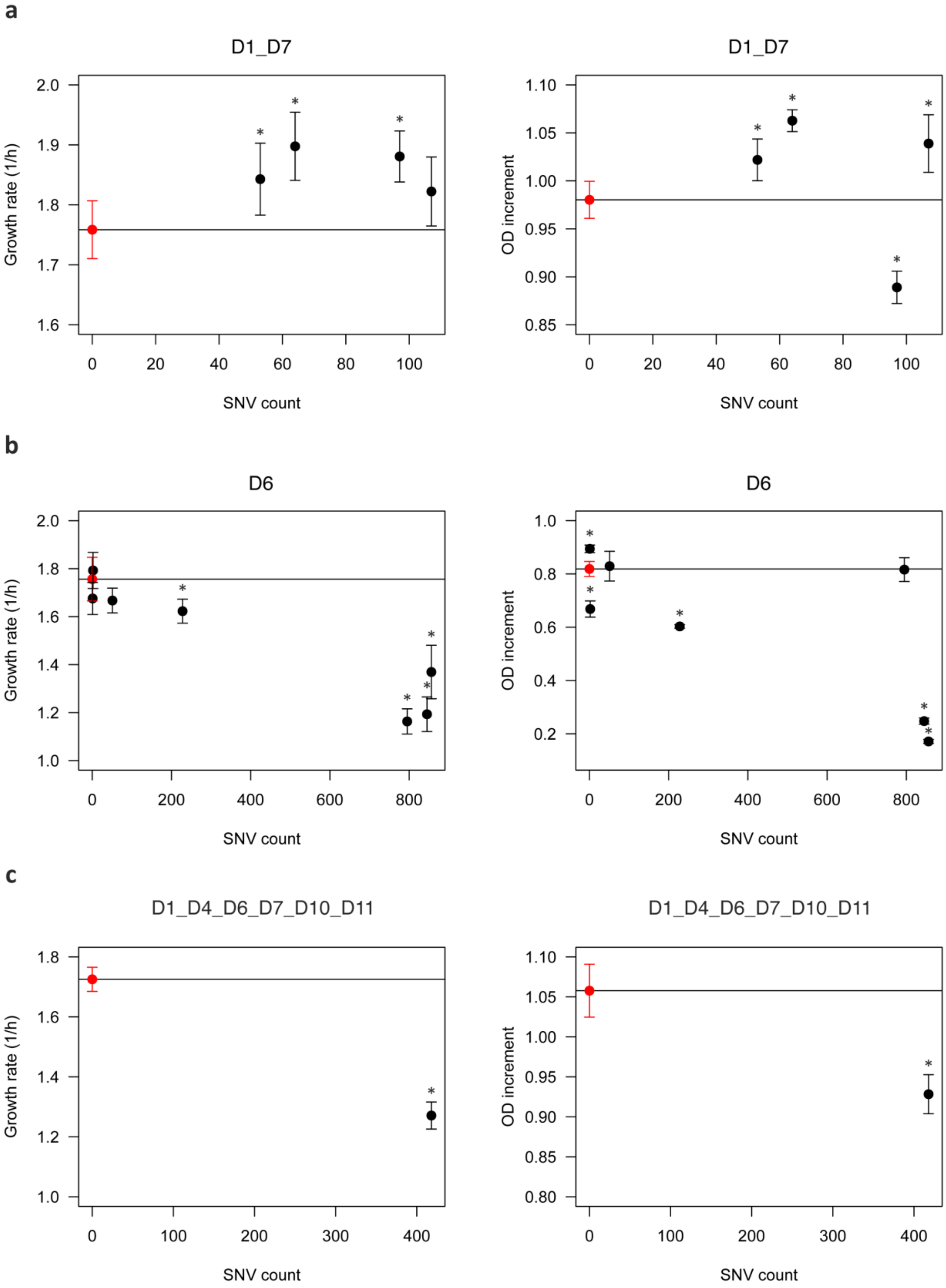
a
D1_D7
Growth rate (1/h)
SNV count
D1_D7
OD increment
SNV count
b
D6
Growth rate (1/h)
SNV count
D6
OD increment
SNV count
c
D1_D4_D6_D7_D10_D11
Growth rate (1/h)
SNV count
D1_D4_D6_D7_D10_D11
OD increment
SNV count

Figure S3. Growth rate and OD increment data of a double- (a), a single- (b), and a six-deletion strain (c), measured in LB. Strains are identified by the deletions (D1 to D12) they carry. The single- and the double-deletion strains are represented by several sequenced variants, possessing various numbers of point mutations. Dots show average growth parameters based on at least 5 replicates. The reconstructed strain with zero point mutation is marked with red. Error bars show 95% confidence intervals. Asterisks show significant difference in comparison to the corresponding strain with zero point mutation (Welch's t-test, $P<0.05$).

a

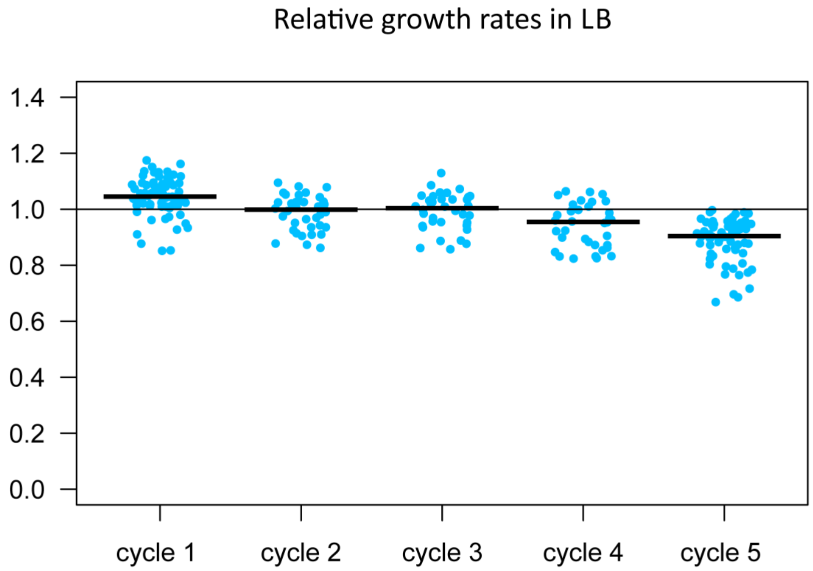

b

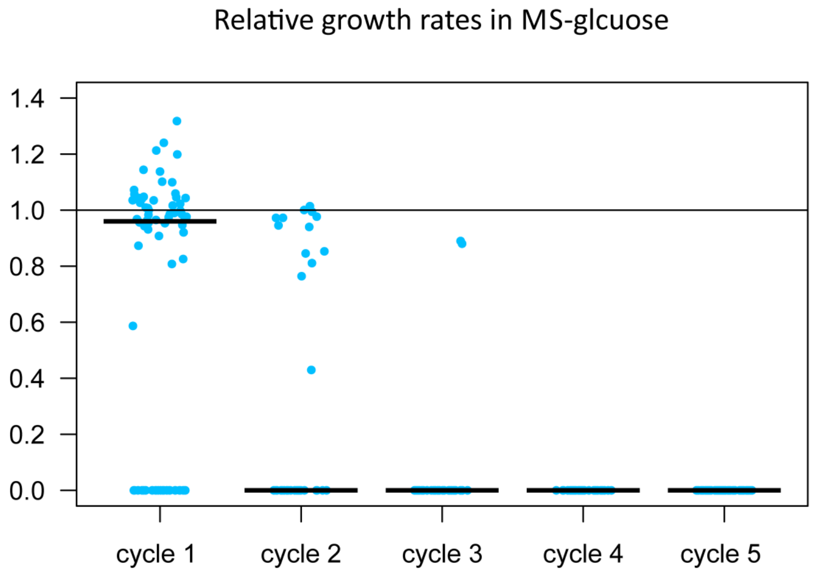

Figure S4. Growth rates of strains obtained in the highly mutagenic protocol. Measurements were carried out both in (a) rich medium (LB) and in (b) minimal medium (MS-glucose). For each cycle, growth of 30 randomly chosen colonies were tested. Growth rates were compared to the average of the parental MG1655. Dots represent the relative growth rate of an individual strain.

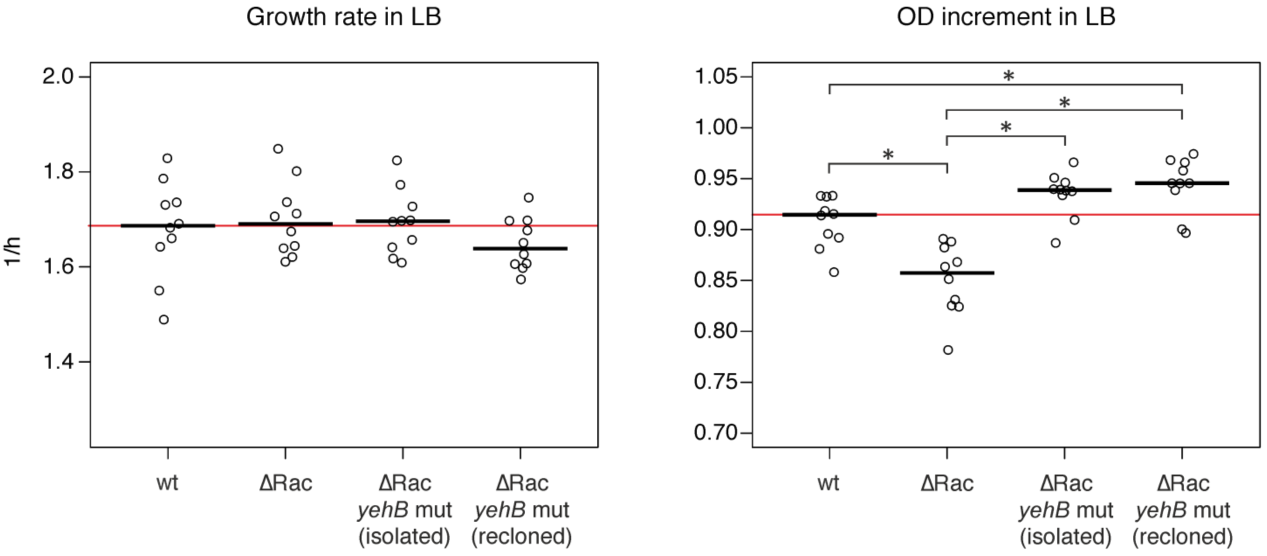

Figure S5. Effect of a compensatory point mutation in the Rac deletion strain: growth parameters of the wild-type and the Rac deletion strain with or without a point mutation in *yehB*. Horizontal black lines show medians of 10 replicate measurements. Asterisks indicate significant difference between strains (P<0.05, one-way ANOVA with post hoc Tukey's test).

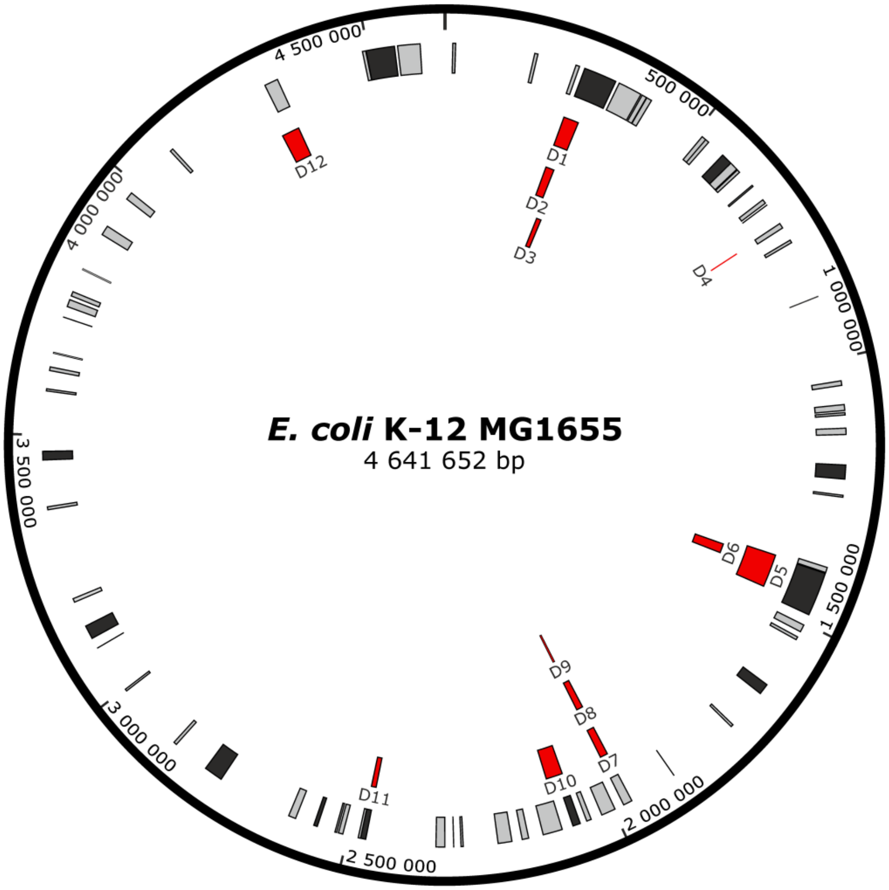

Figure S6. Comparison of deletions obtained by targeted and random methods. Red boxes indicate random deletions obtained in this work (D1 to D12), black boxes represent targeted deletions of the multi-deletion strain MDS12[1], grey boxes show additional deletions of MDS69[2].

a

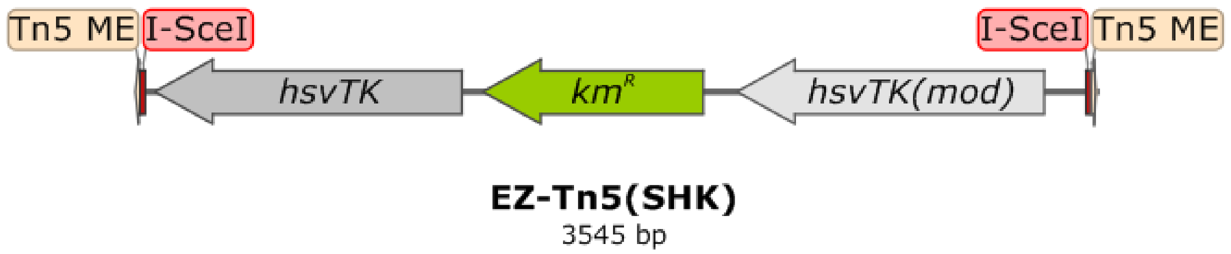

b

```
CTGTCTCTTATACACATCTTAGGGATAACAGGGTAATCGATGCTGTCGGAAACACGGTGCCTGACTGCAAGCTTTCAG
TTAGCCTCCCCCATCTCCCGGGCAAACGTGCGCGCCAGGTCGCATATCGTCGGTATGGAGCCGGGGGTGGTGACGTGG
GTCTGGACCATCCCGGAGGTAAGTTGCAGCAGGGCGTCCCGGCAGCCGGCGGGCGATTGGTCGTAATCCAGGATAAAG
ACGTGCATGGAACGGAGGCGTTTGGCCAAGACGTCCAAGGCCCAGGCAAACACGTTATACAGGTCGCCGTTGGGGGCC
AGCAACTCGGGGGCCCGAAACAGGGTAAATAACGTGTCCCCGATATGGGGTCGTGGGCCCGCGTTGCTCTGGGGCTCG
GCACCCTGGGGCGGCACGGCCGTCCCCGAAAGCTGTCCCCAGTCCTCCCGCCACGACCCGCCGCACTGCAGATACCGC
ACCGTATTGGCAAGTAGCCCGTAAACGCGGCGAATCGCAGCCAGCATAGCCAGGTCCAGCCGCTCGCCGGGGCGCTGG
CGTTTGGCCAGGCGGTCGATGTGTCTGTCCTCCGGAAGGGCCCCAAGCACGATGTTGGTGCCGGGCAAGGTCGGCGGG
ATGAGGGCCACGAACGCCAGCACGGCCTGGGGGGTCATGCTGCCCATAAGGTACCGCGCGGCCGGGTAGCACAGGAGG
GCGGCGATGGGATGGCGGTCGAAGATGAGGGTGAGGGCCGGGGGCGGGGCATGTGAGCTCCCAGCCTCCCCCCCGATA
TGAGGAGCCAGAACGGCGTCGGTCACGGCATAAGGCATGCCCATTGTTATCTGGGCGCTTGTCATTACCACCGCCGCG
TCCCCGGCCGATATCTCACCCTGGTCGAGGCGGTGTTGTGTGGTGTAGATGTTCGCGATTGTCTCGGAAGCCCCCAGC
ACCCGCCAGTAAGTCATCGGCTCGGGTACGTAGACGATATCGTCGCGCGAACCCAGGGCCACCAGCAGTTGCGTGGTG
GTGGTTTTCCCCATCCCGTGGGGACCGTCTATATAAACCCGCAGTAGCGTGGGCATTTTCTGCTCCGGGCGGACTTCC
GTGGCTTCTTGCTGCCGGCGAGGGCGCAACGCCGTACGTCGGTTGCTATGGCCGCGAGAACGCGCAGCCTGGTCGAAC
GCAGACGCGTGTTGATGGCCGGGGTACGAAGCCATGGCTAGTATTTCTCCTCTTTCTCTAGTAGTGCTCAGTATCTCT
ATCACTGATAGGGATGTCAATCTCTAAGCTAATTATTAGAAAAATTCATCCAGCATCAGATGAAATTGCAGTTTGTTC
ATATCCGGATTATCAATGCCATATTTCTGAAACAGACGTTTTTGCAGGCTCGGGCTAAATTCGCCCAGGCAGTTCCAC
AGAATGGCCAGATCCTGATAACGATCCGCAATGCCCACACGGCCCACATCAATGCAGCCAATCAGTTTGCCTTCATCG
AAAATCAGGTTATCCAGGCTAAAATCGCCGTGGGTCACCACGCTATCCGGGCTAAACGGCAGCAGTTTATGCATTTCT
TTCCACACCTGTTCCACCGGCCAGCCGTTACGTTCATCATCAAAATCGCTCGCATCCACCAGGCCGTTGTTCATACGG
CTCTGCGCCTGGGCCAGACGAAACACACGATCGCTGTTAAACGGGCAGTTGCACACCGGAATGCTATGCAGACGACGC
AGAAACACGGCCAGCGCATCCACAATGTTTTCGCCGCTATCCGGATATTCTTCCAGCACCTGAAACGCGGTTTTGCCC
GGAATCGCGGTGGTCAGCAGCCACGCATCATCCGGGGTGCGAATAAAATGTTTAATGGTCGGCAGCGGCATAAATTCG
GTCAGCCAGTTCAGACGCACCATTTCATCGGTCACATCGTTCGCCACGCTGCCTTTGCCATGTTTCAGAAACAGTTCC
GGCGCATCCGGTTTGCCATACAGACGATAAATGGTCGCGCCGCTCTGACCCACGTTATCACGCGCCCATTTATAGCCA
TACAGATCCGCATCCATGTTGCTGTTCAGACGCGGACGGCTACAGCTCGTTTCACGCTGAATATGGCTCATAACACCC
CTTGTATTACTGTTTATGTAAGCAGACAGTTTTATTGTTCATGATGATATATTTTTATCTTGTGCAATGTAACATCAG
AGATTTTGAGACACAAGCGATGATAAGCTGTCCTCGAGTTAATTCGCTTCACCCATTTCACGTGCGAAGGTACGGGCA
AGATCACAGATGGTCGGGATACTCCCAGGCGTCGTCACATGCGTTTGCACCATGCCGCTAGTCAGTTGAAGCAGCGCA
TCACGACAGCCTGCTGGGCTTTGATCATAATCCAGAATAAAAACATGCATGCTGCGCAGACGCTTAGCCAGAACATCC
AGCGCCCACGCGAAAACATTGTACAGATCCCCATTTGGTGCCAGCAGCTCCGGCGCACGGAACAGGGTGAACAGGGTA
TCACCAATGTGCGGACGCGGACCTGCGTTAGACTGAGGTTCCGCACCCTGCGGAGGAACTGCCGTACCGCTCAGTTGA
CCCCAATCTTCACGCCAACTGCCACCGCATTGCAGGTAACGGACAGTGTTCGCCAGCAGGCCATATACACGACGAATC
```

GCTGCCAGCATCGCCAGATCCAGGCGTTCACCAGGACGTTGACGTTTCGCCAGACGGTCGATATGACGATCTTCTGGC
AGCGCCCCCAGCACGATATTGGTCCCAGGCAGAGTAGGCGGAATCAGTGCAACGAATGCCAGGACGGCCTGTGGGGTC
ATAGAACCCATCAGATAACGTGCAGCAGGATAGCACAGCAGAGCCGCAATCGGGTGACGATCAAAAATCAGGGTCAGG
GCAGGCGGCGGCGCATGGCTACTGCCGGCTTCACCACCAATATGCGGCGCCAGAACCGCATCGGTTACTGCGTAAGGC
ATACCCATCGTGATTTGCGCACTGGTCATCACTACGGCTGCATCACCGGCGCTGATTTCCCCTTGGTCCAGACGATGC
TGGGTCGTATAGATATTAGCAATAGTTTCACTCGCGCCCAGCACGCGCCAATACGTCATCGGTTCAGGCACATAAACA
ATATCATCACGGCTGCCCAGCGCAACCAGTAATTGGGTCGTGGTGGTTTTACCCATACCATGCGGCCCATCAATGTAT
ACGCGTAACAGGGTCGGCATCTTTTGCTCCGGACGAACCTCAGTGGCCTCCTGCTGACGACGCGGACGCAGTGCGGTA
CGACGATTAGAATGACCACGGCTGCGCGCTGCCTGATCGAAAGCAGATGCATGCTGGTGACCCGGATAGCTCGCCATG
GTGTAATCCTCCTAAAAGTTAAACAAAATTCTAGATATTATAATTGTTATCCGCTCACAAAGCAAATAAATTTTTCAT
GATTTATCGATGCATGAAGCTCGTGTATGGGGCTGACTTCAGGTGGTCTGCGCGTAATCTCTTGTTCAGCGAAGGGAT
TACCCTGTTATCCCTAAGATGTGTATAAGAGACAG

Figure S7. Schematic view (a) and nucleotide sequence (b) of the modified EZ-Tn5 transposon. Tn5 ME: Tn5 transposase recognition sequence (ME=mosaic end); I-SceI: recognition site of I-SceI meganuclease; *km*^{R}: kanamycin resistance gene; *hsvTK*: herpes simplex virus thymidine kinase; *hsvTK(mod):* codon-modified duplicate of *hsvTK*.

Table S1. Single cycle deletions and background SNV numbers in the MDS42-derived strains. Red boxes indicate the presence of a particular deletion.

**Strain**	**Deletion**			**SNV count**
	D13	D14	D15	
c1_MDS42_1				0
c1_MDS42_2				0
c1_MDS42_3				0
c1_MDS42_4				0
c1_MDS42_5				1
c1_MDS42_6				0
c1_MDS42_8				1
c1_MDS42_10				0
c1_MDS42_18				0
c1_MDS42_7				60
c1_MDS42_14				13
c1_MDS42_16				42

Table S2. Features of deletions obtained following one random deletion cycle on MDS42.

Deletion	Left border	Right border	Size (bp)	Description	Supposed mechanism
D13	266 028	301 308	35 281	genomic segment between REP elements (incl. the deletion of lac operon)	recombination between homologous regions of REP23 and REP31
D14	266 090	283 922	17 833	genomic segment between REP elements	recombination between homologous regions of REP23 and REP28
D15	688 716	695 120	6 405	genomic segment between *ybhR* gene and RIP75 element	unknown, no homology around the deletion

Table S3. Growth parameters of the strains selected for sequencing. (a) MG1655-derived strains obtained by the highly mutagenic protocol, (b) MG1655-derived strains obtained by the weakly mutagenic protocol, (c) MDS42-derived strains obtained by the highly mutagenic protocol. Growth rates and OD increments were compared to that of the parental strain. Mean values and standard deviations (SD) are based on at least 4 replicate measurements.

a

Number of cycles	Selection	Strain	Relative growth rate		Relative OD increment	
			Mean	*SD*	Mean	*SD*
1 control cycle	good grower	contr-56	1.080	*0.025*	1.081	*0.019*
		contr-59	1.079	*0.033*	1.090	*0.019*
1 cycle	good grower	c1-16	0.999	*0.029*	0.971	*0.042*
		c1-17	1.019	*0.042*	0.979	*0.009*
		c1-53	1.077	*0.040*	1.061	*0.014*
		c1-30	1.021	*0.035*	1.121	*0.028*
		c1-21	1.024	*0.063*	1.127	*0.020*
		c1-23	1.017	*0.047*	1.086	*0.013*
		c1-24	1.072	*0.054*	1.121	*0.002*
		c1-48	1.026	*0.053*	1.094	*0.024*
		c1-72	1.064	*0.033*	1.088	*0.033*
		c1-29	1.031	*0.065*	1.048	*0.017*
		c1-32	1.017	*0.067*	1.071	*0.029*
	random	c1-5	0.671	*0.014*	0.795	*0.076*
		c1-9	1.009	*0.052*	0.579	*0.047*
		c1-10	0.717	*0.052*	0.253	*0.017*
		c1-11	1.039	*0.064*	0.693	*0.087*
		c1-12	0.724	*0.096*	0.218	*0.039*
		c1-15	0.992	*0.031*	0.994	*0.030*
		c1-6	0.623	*0.076*	0.356	*0.014*
		c1-14	0.840	*0.014*	0.565	*0.012*
		c1-7	0.989	*0.037*	1.065	*0.004*
		c1-8	0.901	*0.017*	0.939	*0.016*
		c1-13	1.039	*0.034*	1.057	*0.004*
2 cycles	good grower	c2-31	1.058	*0.055*	0.965	*0.036*
		c2-17	0.988	*0.006*	1.109	*0.009*
		c2-36	1.035	*0.028*	1.085	*0.012*
		c2-23	1.112	*0.041*	1.152	*0.073*
		c2-11	1.025	*0.023*	1.121	*0.050*
		c2-8	1.083	*0.023*	0.968	*0.038*
		c2-12	1.063	*0.020*	1.102	*0.022*
		c2-20	1.030	*0.044*	1.092	*0.060*
		c2-26	1.086	*0.025*	1.120	*0.020*
		c2-32	1.107	*0.049*	0.947	*0.052*
5 cycles	good grower	c5-158	0.841	*0.058*	1.042	*0.028*
		c5-170	0.962	*0.044*	1.134	*0.035*

b

Number of cycles	Selection	Strain	Relative growth rate		Relative OD increment	
			Mean	*SD*	Mean	*SD*
1 cycle	good grower	c1_lowmut-80	0.997	*0.049*	1.028	*0.042*
		c1_lowmut-87	1.055	*0.041*	0.763	*0.045*
	random	c1_lowmut-51	0.986	*0.036*	1.030	*0.028*
		c1_lowmut-52	0.968	*0.035*	1.024	*0.015*
		c1_lowmut-53	0.989	*0.027*	1.031	*0.027*
		c1_lowmut-54	0.998	*0.026*	1.040	*0.026*
		c1_lowmut-56	0.966	*0.039*	1.052	*0.028*
		c1_lowmut-57	1.041	*0.022*	0.762	*0.025*
		c1_lowmut-58	0.955	*0.031*	0.989	*0.032*
		c1_lowmut-59	0.981	*0.017*	1.026	*0.035*
		c1_lowmut-55	0.989	*0.025*	1.044	*0.017*
		c1_lowmut-60	0.974	*0.024*	1.017	*0.033*

c

Number of cycles	Selection	Strain	Relative growth rate		Relative OD increment	
			Mean	*SD*	Mean	*SD*
1 cycle	random	c1_MDS42_1	0.995	*0.001*	1.051	*0.067*
		c1_MDS42_2	1.005	*0.015*	1.052	*0.091*
		c1_MDS42_3	1.008	*0.033*	1.074	*0.053*
		c1_MDS42_4	0.992	*0.019*	1.017	*0.005*
		c1_MDS42_5	1.003	*0.027*	1.006	*0.045*
		c1_MDS42_6	0.990	*0.010*	1.049	*0.070*
		c1_MDS42_8	0.995	*0.017*	1.017	*0.010*
		c1_MDS42_10	0.993	*0.020*	1.053	*0.040*
		c1_MDS42_18	0.973	0.017	1.004	0.041
		c1_MDS42_7	0.963	*0.017*	0.985	*0.023*
		c1_MDS42_14	0.952	*0.016*	1.028	*0.024*
		c1_MDS42_16	0.982	*0.012*	1.071	*0.016*

Table S4. Strains and plasmids used in this study.

Strain or plasmid	Relevant characteristics	Function	Source or reference
E. coli K-12 strains:			
MG1655	wild-type	parental strain for generating random deletions	[3]
MDS42	multideletional derivative of wild-type MG1655	parental strain for generating random deletions; host of pST76-A derivatives	[4]
MDS42π	MDS42 strain expressing Pir protein enabling the maintenance of plasmids with R6K γ origin of replication	host of pSG76-A and pSG76-A-Tn5-SHK plasmids	laboratory stock; [5]
Plasmids:			
pHKH	carrying a selection marker (km^R) between the two counterselection genes (*hsvTK)*	source for selection-counterselection cassette of EZ-Tn5(SHK)	[6]
pSG76-A	carrying ap^R and R6K γ-ori, requires Pir protein for replication	plasmid backbone for creating EZ-Tn5(SHK)	[7]
pSG76-A-Tn5-SHK	pSG76-A harbouring ap^R and EZ-Tn5(SHK) transposon	source for EZ-Tn5(SHK) transposon DNA	this study
pSTAST	low-copy number plasmid carrying a temperature-sensitive replicon; expressing I-SceI under the control of the tet promoter; derivative of pSTKST, where km^R is replaced with ap^R	providing I-SceI enzyme upon induction with anhydrotetracycline (aTc)	laboratory stock; [1]
pST76-A derivatives	suicide plasmid with temperature-sensitive replicon and ap^R	generation of targeted, re-constructed deletions	[7]

Supplementary references

1. Kolisnychenko, V. *et al.* Engineering a Reduced Escherichia coli Genome. *Genome Res.* **12**, 640–647 (2002).
2. Karcagi, I. *et al.* Indispensability of Horizontally Transferred Genes and Its Impact on Bacterial Genome Streamlining. *Mol. Biol. Evol.* **33**, 1257–1269 (2016).
3. Blattner, F. R. *et al.* The complete genome sequence of Escherichia coli K-12. *Science* **277**, 1453–1462 (1997).
4. Pósfai, G. *et al.* Emergent properties of reduced-genome Escherichia coli. *Science* **312**, 1044–1046 (2006).
5. Platt, R., Drescher, C., Park, S.-K. & Phillips, G. J. Genetic System for Reversible Integration of DNA Constructs and lacZ Gene Fusions into the Escherichia coli Chromosome. *Plasmid* **43**, 12–23 (2000).
6. Tominaga, M. *et al.* Liquid-Based Iterative Recombineering Method Tolerant to Counter-Selection Escapes. *PLoS ONE* **10**, (2015).
7. Pósfai, G., Koob, M. D., Kirkpatrick, H. A. & Blattner, F. R. Versatile insertion plasmids for targeted genome manipulations in bacteria: isolation, deletion, and rescue of the pathogenicity island LEE of the Escherichia coli O157:H7 genome. *J. Bacteriol.* **179**, 4426–4428 (1997).
